# Can There Be Suppuration without the Presence of Micro Organisms?

**Published:** 1887-02

**Authors:** 


### CAN THERE BE SUPPURATION WITHOUT THE PRESENCE OF MICRO-ORGANISMS?

No greater indication of the progress of pathological knowledge can be cited than the fact that all of our text-books regard suppuration as a stage of the process of inflammation, and so teach, and yet, to-day, no really intelligent pathologist holds this view. Since the issue of the latest text-books, sufficient advance has been made to lead to the positive conclusion that every case of suppuration is due to a fungus which has obtained entrance to the disturbed territory and there proliferated. Clinical experience had established the probability that, if an aseptic condition could be maintained, there would be no gatherings of pus. But abscesses were known in which no bacteria could be found, and certain irritants were introduced into the tissues under what was thought to be aseptic conditions, yet an abscess resulted. These things for a time staggered the advocates of the modern theories. Yet as Tyndall finally demonstrated the source of the contamination in the experiments of Bastian, who thought he had demonstrated the possibility of spontaneous generation, so the sources of error in the earlier experiments in producing suppuration have been shown.

Klemperer, of Berlin, made a series of observations based upon one hundred experiments, and showed that without some infection the exudation, when irritants were used, was always fibrinous and never purulent. Since then other experimenters have verified his conclusions, and lately, in this country, the following experiment has been repeatedly performed, always with the same results. Croton oil, or turpentine, was enclosed in a glass capsule which was hermetically sealed, the whole being carefully sterilized. This was then introduced beneath the skin and within the tissues, and allowed to remain there until the wound was so far closed as to positively indicate that no suppuration existed. The capsule was then broken beneath the skin, and without exposure. In no case was there any purulent discharge. But immediately upon infection, suppuration commenced. It may therefore be considered as established, that without the presence of bacteria there can be no abscess or suppuration.

